# Oral health-related quality of life and dental treatment need 5 to 10 years after hematopoietic cell transplantation

**DOI:** 10.1007/s00520-026-10685-z

**Published:** 2026-04-20

**Authors:** Stephanie J. M. van Leeuwen, Lucky L. A. van Gennip, Laura van Swam, Marijke W. J. van de Ven, Nicole M. A. Blijlevens, Marie-Charlotte D. N. J. M. Huysmans

**Affiliations:** 1 Department of Dentistry, Radboud University Medical Center, Nijmegen, The Netherlands; 2 Department of Hematology, Radboud University Medical Center, Nijmegen, The Netherlands

**Keywords:** Hematopoietic cell transplantation, Oral health-related quality of life, Dental extractions, Dental restorations

## Abstract

**Purpose:**

To investigate the long-term impact of hematopoietic cell transplantation (HCT) on oral health-related quality of life and dental treatment needs 5–10 years post-HCT.

**Methods:**

This study combined questionnaires with a retrospective analysis of dental records. Adult survivors (219) of Radboudumc HCT-treatment in 2012–2017 received OHIP-14, summated XI, MFIQ and OES questionnaires. If returned with informed consent, dental records were requested from the dentist and the number of extractions and restorations performed from 7 years before to 10 years post-HCT was counted. Negative binomial and logistic regression models, adjusted for confounders, were used to evaluate the impact of HCT type (autologous vs. allogeneic) on questionnaire scores, extractions, and restorations.

**Results:**

A total of 169 HCT recipients (84 allogeneic) completed questionnaires, and dental records were available for 145 patients. Allogeneic HCT recipients had significantly higher sum scores for the OHIP, XI, and MFIQ questionnaires (OHIP IRR = 2.1 [95% CI 1.4, 3.3]; XI IRR = 1.17 [95% CI 1.03, 1.34]; MFIQ IRR = 2.6 [95% CI 1.5, 4.5], respectively), but no significant difference was found for the OES. Allogeneic HCT recipients were more likely to have one or more extractions (OR = 4.5 [95% CI 1.3, 17.7]) compared to autologous HCT recipients. No significant difference was observed in the number of restorations between the two groups.

**Conclusion:**

Allogeneic HCT recipients reported worse oral health-related quality of life, more xerostomia complaints, and more mandibular-function complaints than autologous HCT recipients. They were also more likely to undergo extractions post-HCT but no significant difference in restorations was observed.

**Supplementary Information:**

The online version contains supplementary material available at 10.1007/s00520-026-10685-z.

## Introduction

Hematopoietic cell transplantation (HCT) is a potentially curative treatment for various hematological disorders and malignancies. Although HCT has significantly improved survival rates, it can lead to long-term complications affecting multiple organs, including the oral cavity [1, 2]. Oral complications such as chronic graft-versus-host disease (cGVHD), xerostomia, and hyposalivation, which may increase the risk of dental caries, can significantly impact patients’ quality of life post-transplantation [3–6].

The type of HCT, whether autologous or allogeneic, along with the conditioning intensity, can affect the severity and duration of oral complications. At a median of 339 days after allogeneic HCT, over 90% of the survivors experienced oral complications, including xerostomia, cGVHD, oral pain and taste changes [7]. A prospective multinational study found that xerostomia complaints were transient in nature in autologous HCT recipients, but remained elevated in allogeneic HCT recipients 12 months post-transplantation [8].

Little is known about oral health-related quality of life post-HCT. A survey among patients with hemato-oncological diseases reported a relatively good oral health-related quality of life, with most patients having been diagnosed more than two years earlier and having received treatment [9]. In a cross-sectional study among 88 HCT recipients 6 months–6 years post-HCT only a minority of the patients indicated that oral complications significantly impacted their oral health-related quality of life [10]. Since little is known about long-term oral health-related quality of life post-HCT, this study aimed to investigate oral health-related quality of life 5–10 years post-HCT and its association with the type of HCT. In addition, dental treatment need was determined in the years before and after HCT, and its association with the type of HCT.

## Materials and methods

All adult post-autologous or allogeneic HCT survivors treated at the Radboud University Medical Center, Nijmegen, The Netherlands between 2012 and 2017 were invited for this research. The eighty-two patients who were already enrolled in the prospective ORASTEM/HOME-(2) study were excluded [5, 11]. Eligible patients were contacted by telephone to obtain informed consent and to confirm their current address. Patients who did not give informed consent or who could not be reached after multiple calls were excluded. The local institutional review board stated that no formal approval was necessary since this research was not subject to the law concerning research involving human subjects (file number 2022–15858). The study was conducted following the Declaration of Helsinki and in accordance with Good Clinical Practice guidelines.

### Oral health-related quality of life

Oral health-related quality of life was determined using the Dutch version of the validated Oral Health Impact Profile OHIP-14 questionnaire consisting of 14 questions answered on a 5-point scale ranging from (0) never to (4) very often [12]. Xerostomia was measured using the validated Summated Xerostomia inventory (XI) consisting of 5 questions answered on a 3-point scale ranging from (1) never to (3) always [13]. The mandibular function was measured using the first nine questions from the Mandibular Function Impairment Questionnaire (MFIQ) questionnaire [14]. The answers were scored on a 5-point scale ranging from (0) never to (4) very often. The orofacial esthetic perception was measured using the validated Orofacial Esthetic Scale (OES) questionnaire consisting of 8 questions answered on a 0–10 scale (dissatisfied–very satisfied) [15]. Next to those questionnaires, four additional questions were asked about patients’ own perception of their oral health post-HCT, whether or not they have the impression that their treatment need had increased and whether they suffer from actual cGvHD (in allogeneic HCT recipients only).

These questionnaires were sent on paper to all patients between June 2023 and October 2023. No reminders were sent. With the questionnaires, all patients were also sent a form where they could fill in their current dentist and give consent to obtain dental records from their dentist. After consent, the dental records were asked from their treating dentist. In case of no response from the dental office, a reminder was sent after a few weeks.

### Medical records

Patient characteristics, medical diagnosis, transplant and treatment details were extracted from the electronic patient records of the hematology department (Radboudumc).

### Dental records

From the dental records the number of natural teeth, excluding third molars, around the date of answering the questionnaires was determined. Counting from the HCT date, the number of years with dental visits was determined from the dental records. A year was included only if entries for routine check-ups or oral hygiene sessions were present; otherwise, that year was not counted as having visited the dentist. The number of restorations and extractions was noted in the period from 7 years before HCT (excluding the year before HCT) to 10 years after HCT. For restorations the billing-codes V15, V71, V72, V73, V74, V81, V82, V83, V84, V91, V92, V93 and V94 were counted. For extractions the billing-codes H11, H16 and H35 were counted and the files were searched for mentions of referrals to an oral surgeon, to include also the more complicated extractions performed by the oral surgeon. Extractions or restorations performed in third molars were excluded.

### Data analysis

All analyses were conducted in R (4.4.1, R Foundation for Statistical Computing, Vienna, Austria). For each completely filled-in questionnaire, the sum of all questions was calculated. The higher the total OHIP, XI and MFIQ-score, the lower the oral health-related quality of life, the more xerostomia and mandibular function complaints, respectively. For the OES questionnaire, the reverse applies: the lower the score, the lower the perceived esthetics. Time between HCT and answering the questionnaires was estimated using a reference date in the middle of the distribution period (the first of August 2023).

Due to non-normal distributions, negative-binomial models were used to assess the effect of the type of HCT (autologous versus allogeneic) on the sum score of the questionnaires, corrected for potential confounders including age, sex, number of natural teeth and time after transplant (in years). Since a dental record to count the number of teeth could not be obtained from every patient with completely answered questionnaires (15% was missing), the number of teeth was imputed using predictive mean matching in 50 datasets. The results of the analysis were subsequently pooled across all imputed datasets using the Rubin’s rules [16].

The number of extractions and restorations per year for each patient before and after HCT was calculated by dividing the total number of extractions and restorations by the number of years with dental visits. To obtain the most reliable numbers, we selected only those patients for whom we had dental record information for at least four years before and four years after HCT. Due to the low number of extractions, the extractions post-HCT were dichotomized into none and one or more. This was subsequently used as the dependent variable in logistic regression models with type of HCT as the independent variable and the extractions per year pre-HCT as a covariate. The logistic regression model was additionally adjusted for potential confounders, including age, sex, number of natural teeth and number of years with dental visits post-HCT. The association between type of HCT and number of restorations post-HCT was determined using negative binomial models with the number of years the dentist was visited post-HCT as the offset variable. The number of restorations per year pre-HCT was added as a covariate in the crude and adjusted models. The model was additionally adjusted for potential confounders including age, sex and number of natural teeth. For all regression models the Incidence Rate Ratios (IRR; negative binomial models) or Odds Ratios (OR; logistic regression) and accompanying 95% confidence intervals (CIs) were visualized in dot-and-whisker plots of the crude and adjusted models.

The correlation between oral health-related quality of life and extractions and restorations per year was graphically visualized using scatterplots.

## Results

**Fig. 1:**
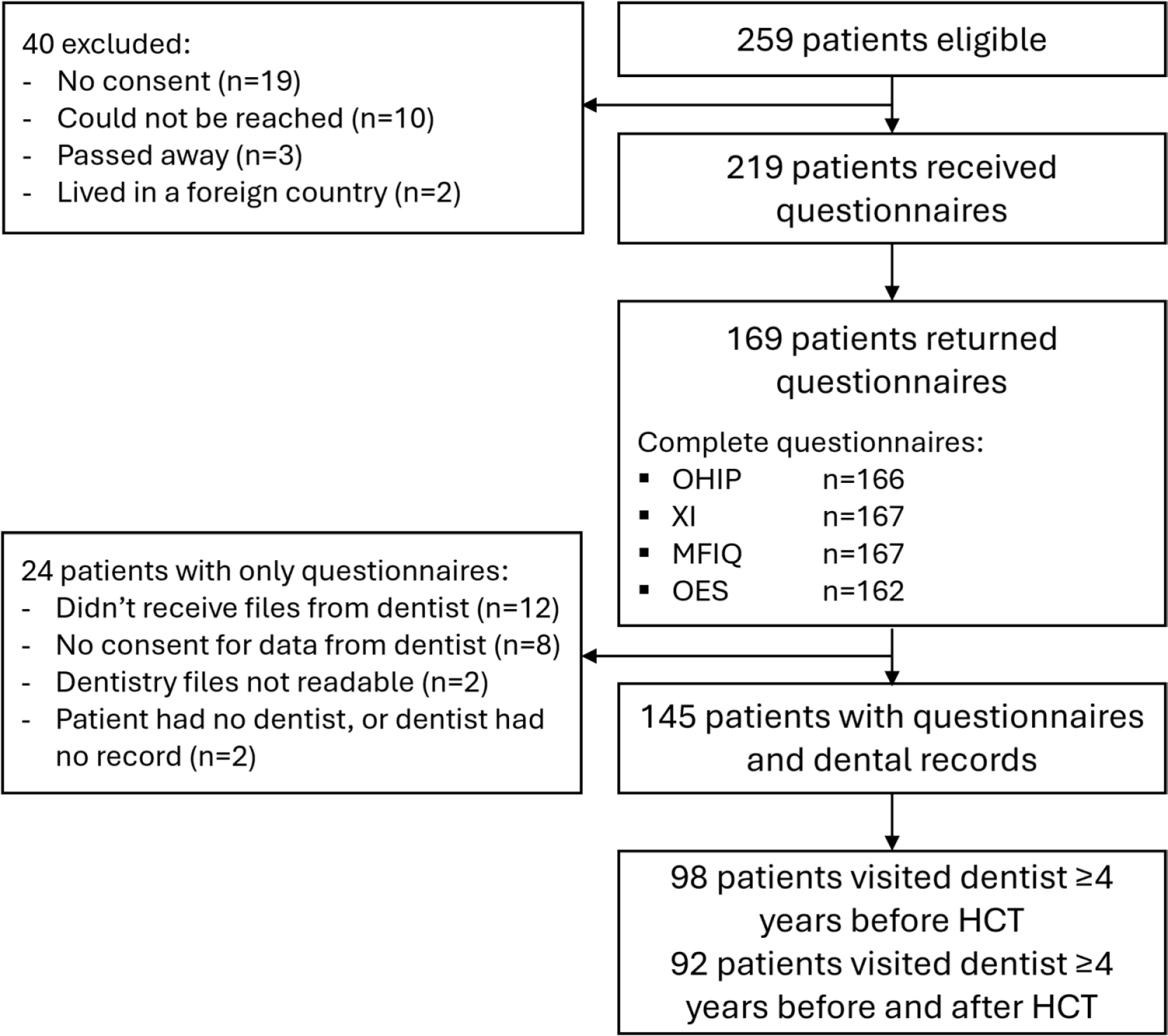
Flow chart of patient selection. OHIP: Oral Health Impact Profile (OHIP-14) questionnaire, XI: Xerostomia inventory, MFIQ: Mandibular Function Impairment Questionnaire, OES: Orofacial Esthetic Scale, HCT: Hematopoietic cell transplantation

In total, 259 patients were eligible and 219 questionnaires were sent out, of which 169 (77%) questionnaires were returned (Fig. 1). Baseline characteristics of all patients are reported in Table 1. Almost all autologous patients received MAC, while most allogeneic patients received non-myeloablative (NMA) conditioning. The time in years post-HCT was higher in the allogeneic patients compared to the autologous patients.

**Table 1:** Patient characteristics

	Total (*n* = 169)	Autologous (*n* = 85)	Allogeneic (*n* = 84)
Age (at HCT)			
Mean	54.4	56.3	52.5
Median	57	59	55
Range	18–71	18–70	19–71
Sex n male (%)	108 (64)	52 (61)	56 (67)
Disease n (%)			
MM	42 (25)	39 (46)	3 (4)
NHL	36 (21)	27 (32)	9 (10)
AML	35 (21)	4 (5)	31 (37)
ALL	15 (9)	-	15 (18)
MDS	15 (9)	1 (1)	14 (17)
Other	26 (15)	14 (16)	12 (14)
Type conditioning therapy n (%)			
NMA	49 (29)	-	49 (58)
RIC	18 (11)	6 (7)	12 (14)
MAC	102 (60)	79 (93)	23 (27)
Ever had diagnosis of cGVHD n (%)	45 (27)	-	45 (54)
Time in years since HCT (ref = 01–08–2023)			
Mean	8.0	7.5	8.5
Median	7.9	6.8	8.6
Range	5.6–11.5	5.6–11.4	5.7–11.5
Second HCT n (%)	9 (5)	4 (5)	5 (6)
Number of natural teeth	*n* = 144	*n* = 75	*n* = 69
Mean	22.8	22.3	23.5
Median	26.0	27.0	26.0
Range	0–28	0–28	0–28

HCT: Hematopoietic cell transplantation, cGVHD: chronic graft-versus-host disease

### Questionnaire scores

**Fig. 2:**
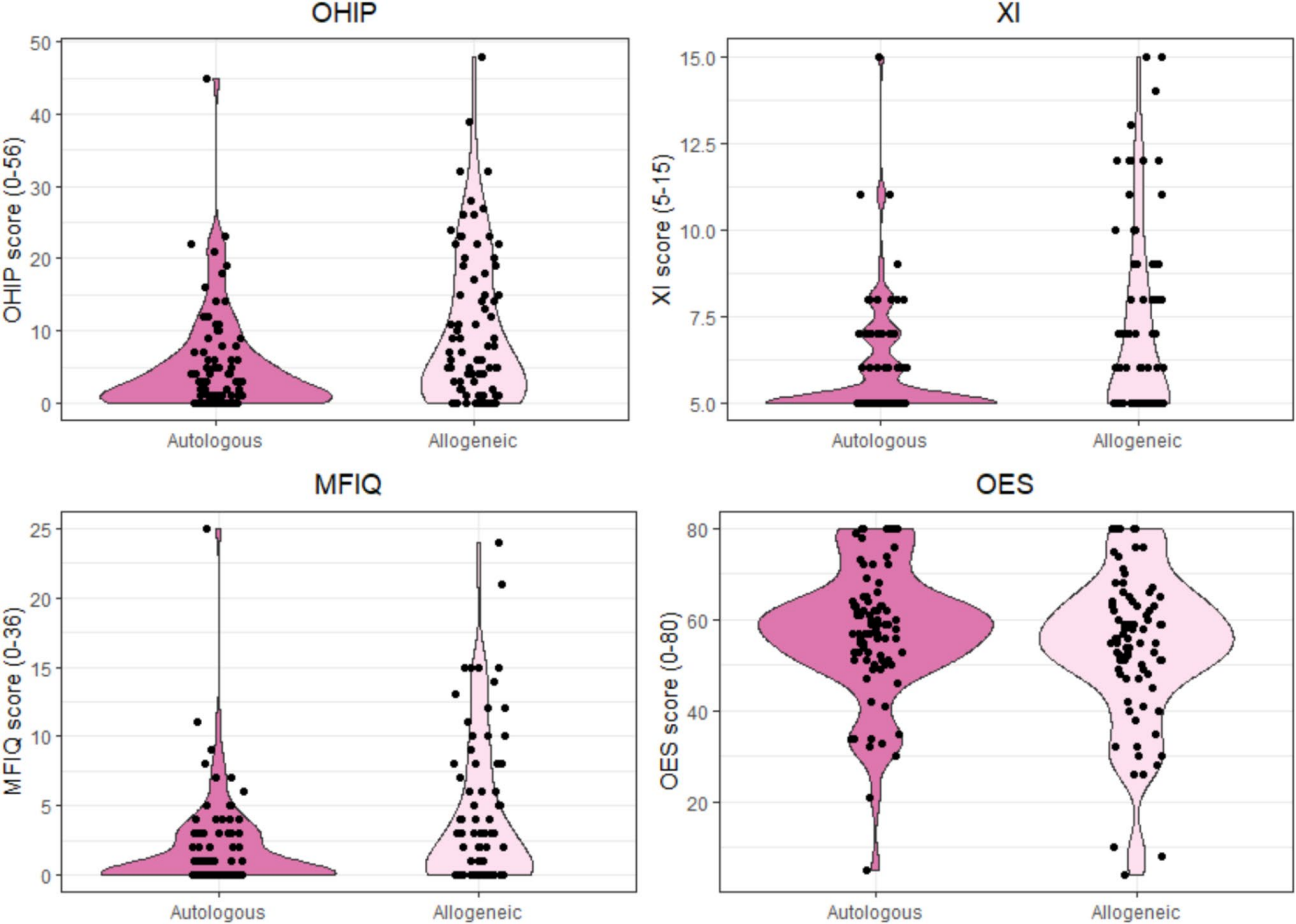
Violin plots of the sum scores of the OHIP, XI, MFIQ and OES questionnaires for autologous and allogeneic HCT recipients. OHIP: Oral Health Impact Profile (OHIP-14) questionnaire, XI: Xerostomia inventory, MFIQ: Mandibular Function Impairment Questionnaire, OES: Orofacial Esthetic Scale, HCT: Hematopoietic cell transplantation

The distribution of total scores for all questionnaires is shown in Fig. 2. Compared to the highest possible score, most OHIP, XI and MFIQ scores were positioned on the lower edge. Especially within the autologous HCT recipients the scores were around 0 or 5 indicating that many patients answered ‘never’ on all questions. Allogeneic HCT recipients showed a wider range of scores. Visual inspection of scatterplots (Supplementary Fig. S1) indicates that patients with higher OHIP scores tended to have higher XI and MFIQ scores and lower OES scores. This pattern was supported by the corresponding Spearman correlations (rho = 0.52, *p* < 0.001; rho = 0.70, *p* < 0.001; rho = –0.44, *p* < 0.001, respectively).

Across individual questionnaire items, no differences between autologous and allogeneic HCT recipients were noticed, except for generally higher scores for the allogeneic HCT recipients (Supplementary Figs. S2–S4). Within the OHIP questionnaire, the highest scores were observed in the domains of physical pain and psychological discomfort. Within the MFIQ questionnaire, the items related to taking a large bite and chewing hard or resistant foods showed the highest scores.

#### Group differences based on regression models

**Fig. 3:**
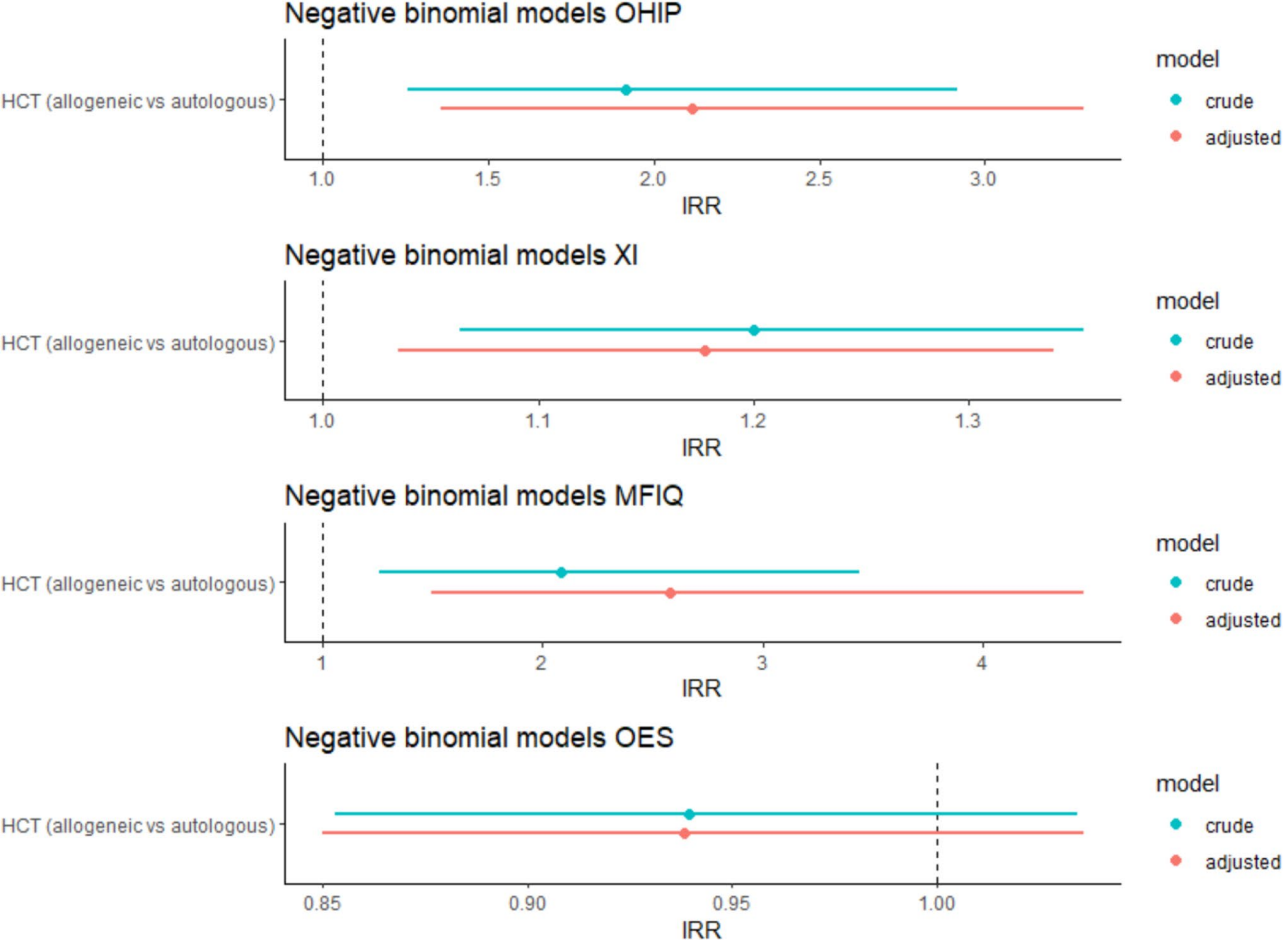
Dot-and-whisker plots showing the Incidence Rate Ratios (IRR) and accompanying 95% confidence intervals (CI) of the crude and adjusted negative binomial models. The adjusted models are adjusted for age, sex, number of natural teeth and time after transplant (in years). OHIP: Oral Health Impact Profile OHIP-14 questionnaire, XI: Xerostomia inventory, MFIQ: Mandibular Function Impairment Questionnaire, OES: Orofacial Esthetic Scale, HCT: Hematopoietic cell transplantation

Figure 3 shows the dot-and-whisker plots with IRRs and accompanying 95% CIs of the crude and adjusted negative binomial models. For the OHIP, XI and MFIQ, the IRRs and 95% CIs were above 1, indicating significantly higher sum score among allogeneic HCT recipients. No significant differences were observed for the OES questionnaire.

#### Variation within allogeneic HCT recipients

Within the allogeneic HCT recipients no clear differences in questionnaire scores are observed based on history of cGvHD, the types of conditioning therapy and TBI dose (Supplementary Figs. S5–S7). We did not test for statistical differences here since it was not in the aim of the study. Twenty allogeneic HCT recipients (23.8%) answered the additionally asked questions that they were currently suffering from oral cGvHD. Those patients scored relatively high on the OHIP, XI and MFIQ questionnaires and low on the OES questionnaire.

### Dental records

Dental records were obtained from 145 (86%) patients (Fig. 1). For 24 patients, no pre-HCT data were available. Six patients were edentulous; two of them became edentulous in the 6th and 11th year post-HCT. In total, 92 (63%) patients had ≥ 4 years of dental record data before and after HCT and were included in the analysis.

Only a limited number of extractions were performed during the study period. The median number of extractions per year was 0 both before and after HCT. Pre-HCT, 20/92 (22%) patients underwent extractions (ranging from 1–12), compared with 29/92 (32%) patients post-HCT (ranging from 1–28). Restorations were performed in 88% of patients pre-HCT and 97% of patients post-HCT. The median number of restorations per year pre-HCT was 0.83 and post-HCT 1.00.

#### Group differences based on regression models

Considering extractions post-HCT, 44% of allogeneic HCT recipients versus 20% of the autologous HCT recipients were affected. After adjusting for age, sex, number of natural teeth, number of extractions per year pre-HCT and years visited the dentist post-HCT, allogeneic HCT recipients were more likely to have one or more extractions post-HCT compared to autologous HCT recipients (OR 4.5 [95% CI 1.3, 17.7]).

**Fig. 4:**
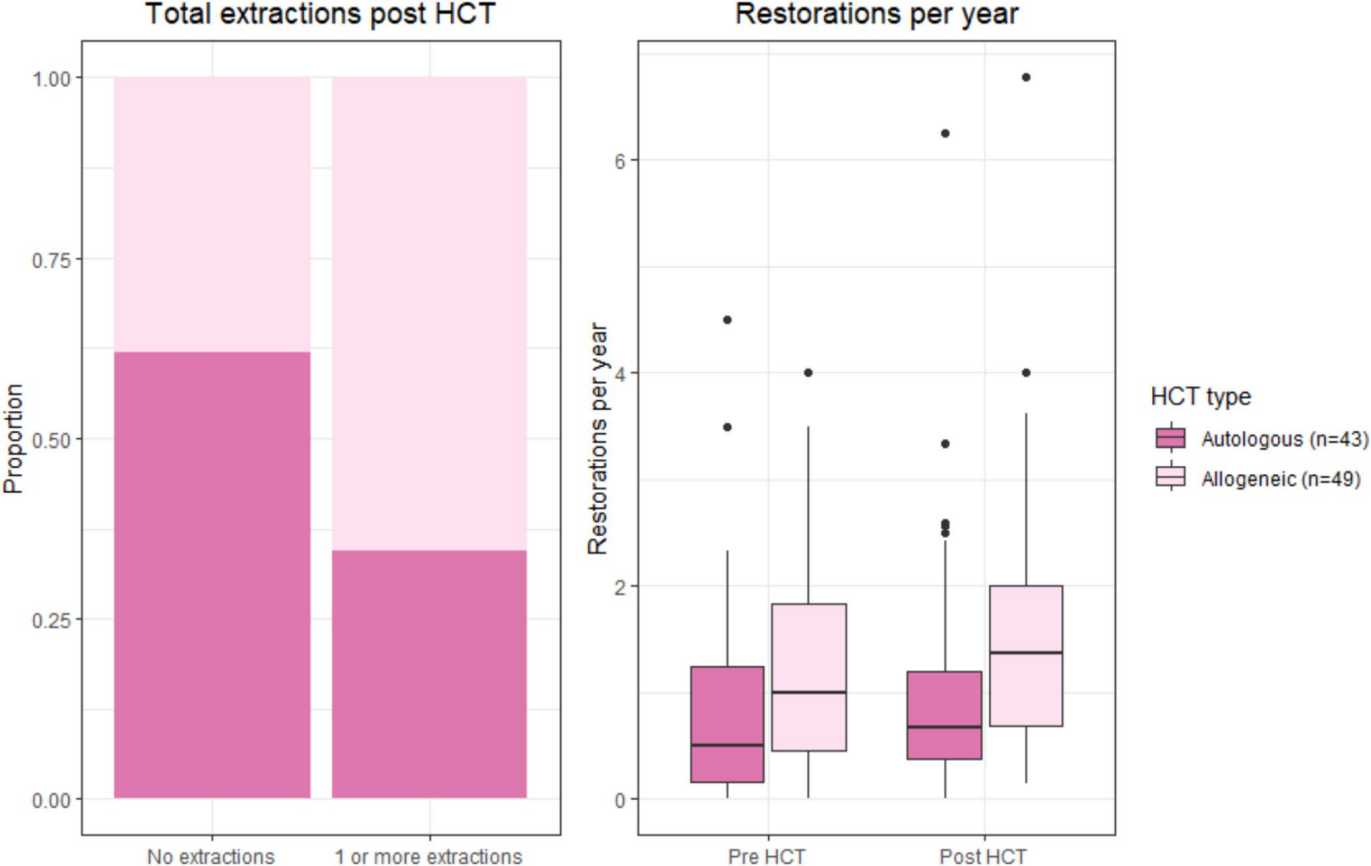
Bar chart with the proportion of total extractions post-HCT for autologous and allogeneic Hematopoietic Cell Transplantation (HCT) recipients and boxplots for the amount of restorations per year before pre- and post-HCT for the autologous and allogeneic HCT recipients

Considering restorations, the median number per year is higher in the allogeneic HCT recipients both before and after HCT (Fig. 4). After adjusting for the number of restorations per year pre-HCT, age, sex and number of natural teeth, allogeneic HCT recipients had 17% more restorations per observation year post-HCT than autologous HCT recipients. This was not statistically significant (IRR 1.17 [95% CI 0.86, 1.58]).

### Correlation between oral health-related quality of life and extractions and restorations per year

Potential associations were explored using visual inspection of scatterplots only. In the period post-HCT, one patient underwent a full-mouth extraction and also experienced a worse oral health-related quality of life with high OHIP, XI and MFIQ scores and low OES scores. After excluding this patient from the scatterplots, we observed graphical indications of a positive linear relationship between OHIP and MFIQ sum scores and the number of extractions per year post-HCT (Supplementary Fig. S8). Visual inspection also suggested a positive relationship between OHIP and XI scores and the number of restorations per year post-HCT (Supplementary Fig. S8).

## Discussion

With the OHIP-14, XI, MFIQ and OES questionnaires we investigated patient-reported oral health-related quality of life 5–10 years post-HCT. Although the oral health-related quality of life scores were generally good 5–10 years post-HCT, allogeneic HCT recipients had a significantly lower oral health-related quality of life after adjusting for confounding. Although there were only a few extractions performed in the years post-HCT, allogeneic HCT recipients were more likely to have one or more extractions compared to autologous patients. For the number of restorations post-HCT we did not find a significant difference between autologous and allogeneic HCT recipients.

The significantly worse oral health-related quality of life of allogeneic HCT recipients is consistent with a publication from 2011 reporting the first year post-HCT. There, allogeneic HCT recipients receiving a MAC conditioning therapy had more symptoms and lower functioning scores compared to the allogeneic patients receiving RIC conditioning therapy and autologous HCT recipients [17]. One year post-HCT, xerostomia and taste changes were increased compared to baseline in the allogeneic HCT recipients receiving a MAC conditioning therapy. The authors hypothesized that this could be due to the use of TBI in this patient population. However, in an earlier prospective longitudinal study up to five years post-HCT from our group we did not observe a significant difference between the allogeneic HCT recipients with or without TBI [5].

In an older study among acute myeloid leukemia patients one year after treatment, cGvHD and TBI were suggested to play a role in the worse quality of life observed in allogeneic bone marrow transplants compared to autologous bone marrow transplants and intensive consolidation chemotherapy [18]. In the study of Andersson et al. (2011) [17] the influence of cGvHD was not investigated, but it was discussed that the role of cGvHD on health-related quality of life was still ambiguous. In the present study we did not observe a difference between the allogeneic HCT recipients ever diagnosed with cGvHD and recipients without a cGvHD diagnosis. However, in the present study we were dependent on the electronic health records for the diagnosis of cGvHD and we have no data on the last status or any given treatments for cGvHD. We found that 20/84 (23.8%) of patients currently suffering from oral cGvHD scored relatively high on the OHIP, XI and MFIQ questionnaires and low on the OES questionnaire.

A literature review about the psychosocial aspects of HCT indicated that allogeneic HCT recipients also had greater impairments in overall quality of life compared to autologous HCT recipients or chemotherapy alone [19]. Acute and chronic GvHD have an adverse impact on quality of life mainly reflected in physical well-being. In a large study, active cGvHD had a negative impact on quality of life, while resolved cGvHD had no impact on quality of life in allogeneic HCT recipients [20]. Especially patients with cGvHD and self-reported depression and anxiety are a vulnerable population for impaired quality of life and poor functioning and HCT-related morbidity [21].

The results of the OHIP-14 and shortened XI questionnaire are in line with the results of the survey study among Dutch patients diagnosed with hemato-oncological diseases, where oral health-related quality of life was rated as rather good as well and the domains physical pain and psychological discomfort were also the highest scoring domains [9]. Moreover, patients with a history of allogeneic HCT reported more xerostomia. In that study, 62.5% of allogeneic HCT patients were qualified as having xerostomia (≥ 8 on S-XI). Using the same threshold, 22.2% (37/167) of patients of the present study were classified as having xerostomia. Within those patients with xerostomia, 73% underwent an allogeneic HCT. Although the treatment history of the patients in the study of Laheij et al. (2024) was diverse, most patients who answered the surveys had their last treatment session more than 2 years ago. A recent report of an ongoing cohort study also found symptoms such as xerostomia and oral pain and a reduced oral health-related quality of life two years after allogeneic HCT [22].

The positive linear relationship between the number of extractions and restorations per year and the OHIP sum scores supports the hypothesis that a low quality of life 5–10 years post-HCT is associated with a higher dental treatment need. In line with this hypothesis we found an increased risk of allogeneic HCT recipients for one or more dental extractions post-HCT after adjusting for confounding. However, we did not observe the same trend for the number of restorations post-HCT. While the association with HCT type was not significant, the number of restorations per year pre-HCT was significantly associated with the number of restorations post-HCT (crude model: IRR 1.6 [1.4; 1.9]; adjusted model: IRR 1.7 [1.4, 1.9]), indicating that the number of restorations was already higher in allogeneic patients pre-HCT. It is unknown if mucosal infiltration, as frequently encountered in acute leukemia [23], might play a role in the worsened oral health status pre-HCT in allogeneic HCT recipients.

Little is known about dental treatment need in HCT recipients. One study found a higher DMFT index indicating a higher dental treatment need four years post-HCT, but according to the authors this might be due to the pre-HCT dental protocols that favored extractions over conservative approaches [24]. In our earlier prospective longitudinal study up to 5 years post-HCT [5] the number of patients having dental restorations was almost similar to the current study, however, the median number of restorations per year is higher in the current study. This may be partially explained by restorations placed on endodontically treated teeth being included in the current study but excluded in the earlier prospective study. When looking at restorations or dental treatment needs in the general population, comparable numbers for mean number of restorations per year were found among adults in the North West of England [25] and adults in Finland between 2012 and 2017 [26].

There are some limitations of this study that should be taken into account for the interpretation of our results. Although the response rate of our study was high, the sample size was relatively large compared with existing literature and we investigated a relatively long follow-up time post-HCT, the monocenter design limits the generalizability of the results. The analysis of dental outcomes was further restricted to patients with sufficient dental records before and after HCT, which may introduce selection bias towards individuals with more regular dental attendance. Moreover, because no clinical oral examinations were performed and current medication use was not collected, the present oral health status and potential medication-related influences on xerostomia could not be fully assessed. Also, dental procedures were used as proxies for treatment need, which do not fully capture the clinical oral condition. In addition, the observed differences between autologous and allogeneic HCT recipients should be interpreted as associations rather than causal relationships. These groups inherently differ in underlying diagnosis, conditioning regimens, and risk of chronic graft-versus-host disease, and these factors were not fully accounted for in the regression models, leaving room for residual confounding.

Although allogeneic HCT recipients were more likely to undergo dental extractions in the years post-HCT, the current study found no substantial increase in overall dental treatment need among HCT recipients 5–10 years post-HCT. For optimal management of oral complications in hemato-oncological diseases and HCT, a multidisciplinary approach, including an oral health professional, is recommended [27]. Monitoring of oral complications should be conducted in all HCT patients, with particular attention to allogeneic HCT recipients exhibiting signs of oral cGvHD.

## Conclusion

Allogeneic HCT recipients reported a lower oral health-related quality of life, more xerostomia complaints and more complaints related to the mandibular function compared to autologous HCT recipients 5–10 years post-HCT. Allogeneic HCT recipients were also more likely to have undergone one or more dental extractions post-HCT, although the number of restorations did not differ between the groups.

## Supplementary Information

Below is the link to the electronic supplementary material.

ESM 1 (DOCX 139 KB)

## Data Availability

The dataset is published in a data acquisition collection of the Radboud Data Repository. Data cannot be made publicly available as participants did not consent to share individual data. The dataset is archived under closed access conditions. Only the metadata of the dataset is published with 10.34973/nh8z-e483.
