# Supplementary material for: Oral health-related quality of life and dental treatment need 5 to 10 years after hematopoietic cell transplantation
Source: Support Care Cancer. 2026 Apr 20;34(5):448. doi: 10.1007/s00520-026-10685-z (PMC13095916; doi:10.1007/s00520-026-10685-z)
Supplement: Supplementary file 1 — (DOCX 139 KB) [file 520_2026_10685_MOESM1_ESM.docx]

**Supplementary Figures**

**Oral health-related quality of life and dental treatment need 5 to 10 years after hematopoietic cell transplantation**

S.J.M. van Leeuwen^1^, L.L.A. van Gennip^1^, L. van Swam^1^, M. van de Ven^1^, N.M.A. Blijlevens^2^, M.C.D.N.J.M. Huysmans^1^

^1^Department of Dentistry, Radboud university medical center, Nijmegen, The Netherlands
^2^Department of Hematology, Radboud university medical center, Nijmegen, The Netherlands

Corresponding author:

S.J.M. van Leeuwen: [stephanie.vanleeuwen@radboudumc.nl](mailto:stephanie.vanleeuwen@radboudumc.nl)

Journal: Supportive Care in Cancer

**
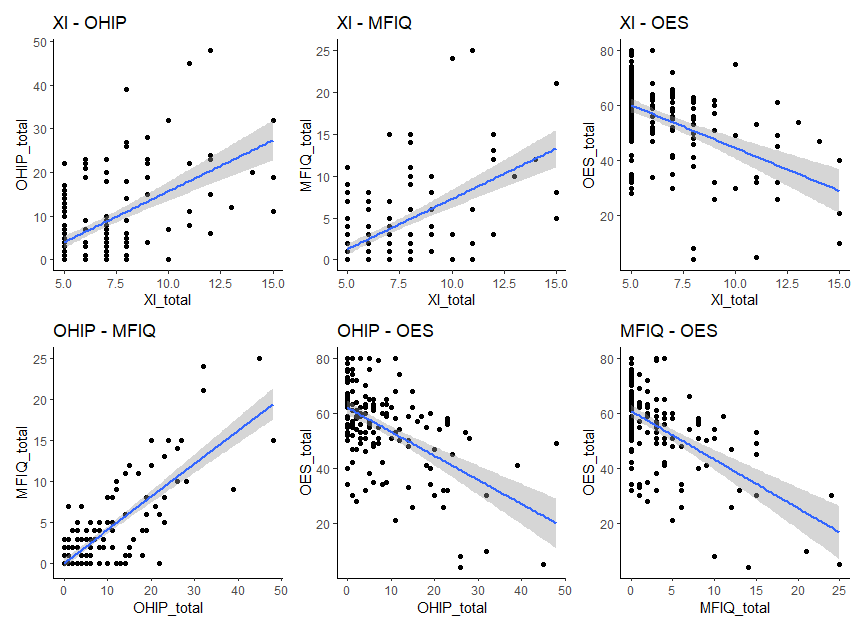
**

**Figure S1.** Scatter dot plots to visualize the correlation between the sum scores of the different questionnaires. Spearman’s rank correlations: XI – OHIP rho = 0.52, p< 0.001; XI – MFIQ rho = 0.50, p< 0.001; XI – OES rho = -0.39, p< 0.001; OHIP – MFIQ rho = rho = 0.70, p< 0,001; OHIP – OES rho = -0.44, p< 0.001 and MFIQ – OES rho = -0.41 p< 0.001.

**
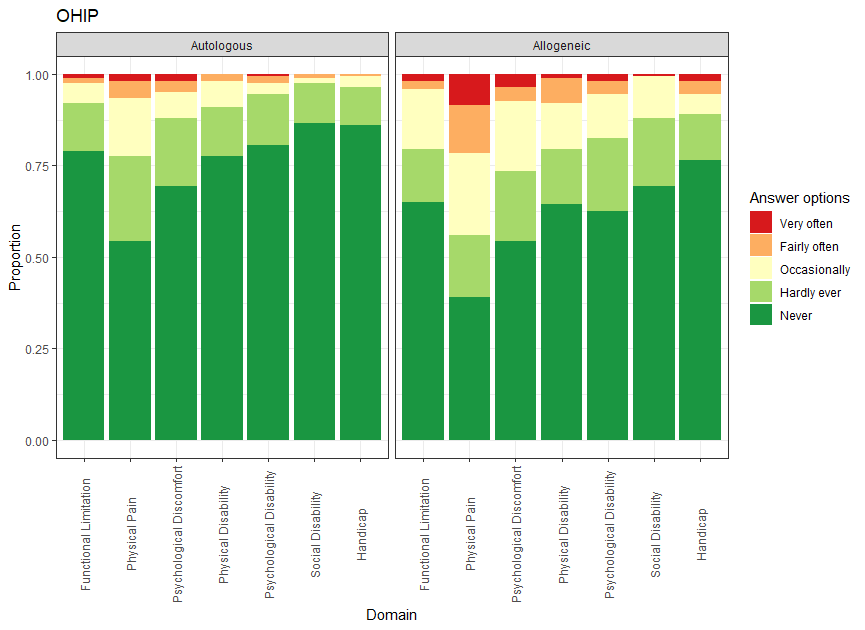
**

**Figure S2.** Detailed visualization of the different answer options over the 7 domains of the OHIP-14 questionnaire for the autologous and allogeneic HCT recipients.

**
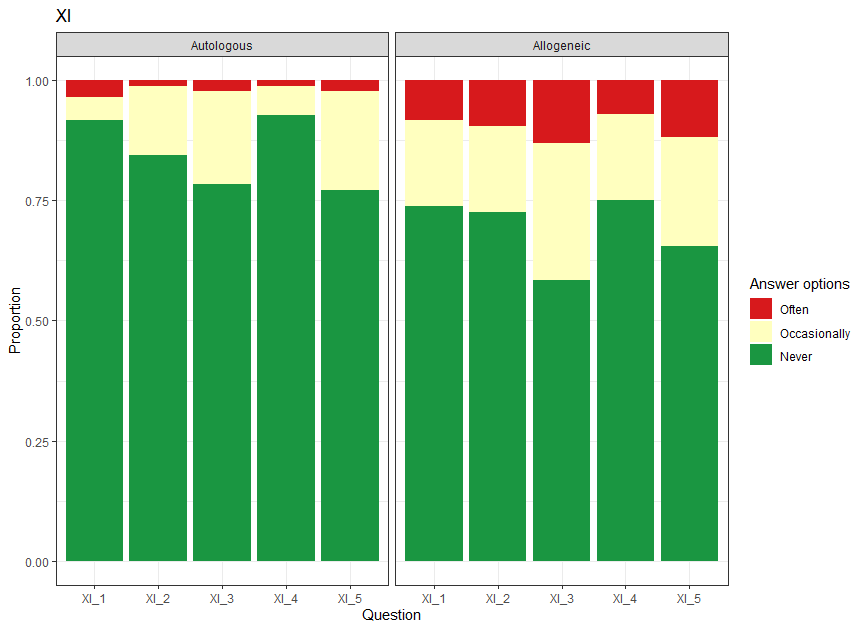
**

**Figure S3.** Detailed visualization of the different answer options of the individual questions of the Shortened XI questionnaire for the autologous and allogeneic HCT recipients.

**
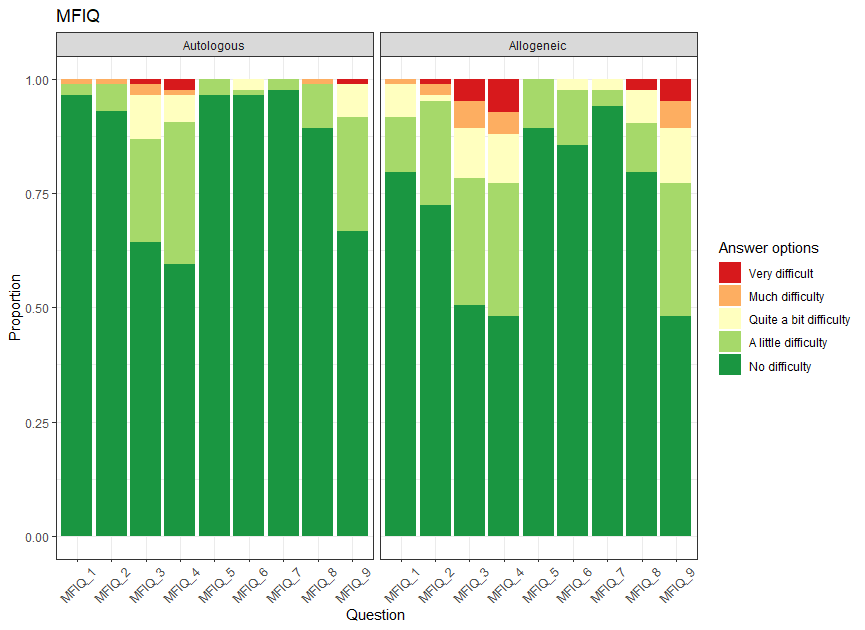
**

**Figure S4.** Detailed visualization of the different answer options of the individual questions of the MFIQ questionnaire for the autologous and allogeneic HCT recipients.

**
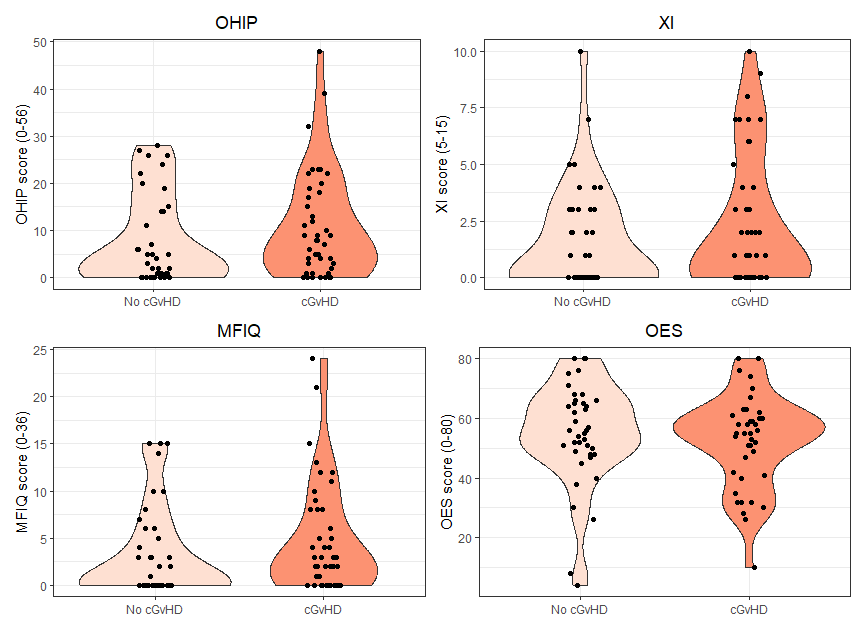
**

**Figure S5.** Violin plots with the sum scores of the OHIP-14, S-XI, MFIQ and OES questionnaires within the allogeneic HCT recipients who had never had a diagnosis of chronic Graft-versus-Host Disease (cGvHD) and those who ever had a diagnosis of cGvHD.

**
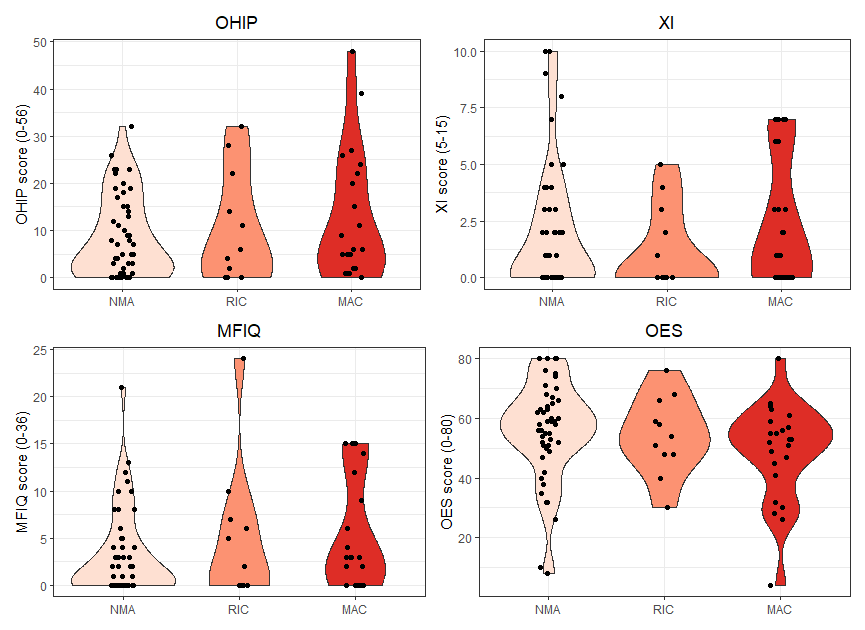
**

**Figure S6.** Violin plots with the sum scores of the OHIP-14, S-XI, MFIQ and OES questionnaires within the allogeneic HCT recipients receiving a non-myeloablative (NMA) conditioning therapy, reduced intesity (RIC) conditioning therapy and myeloablative (MAC) conditioning therapy.

**
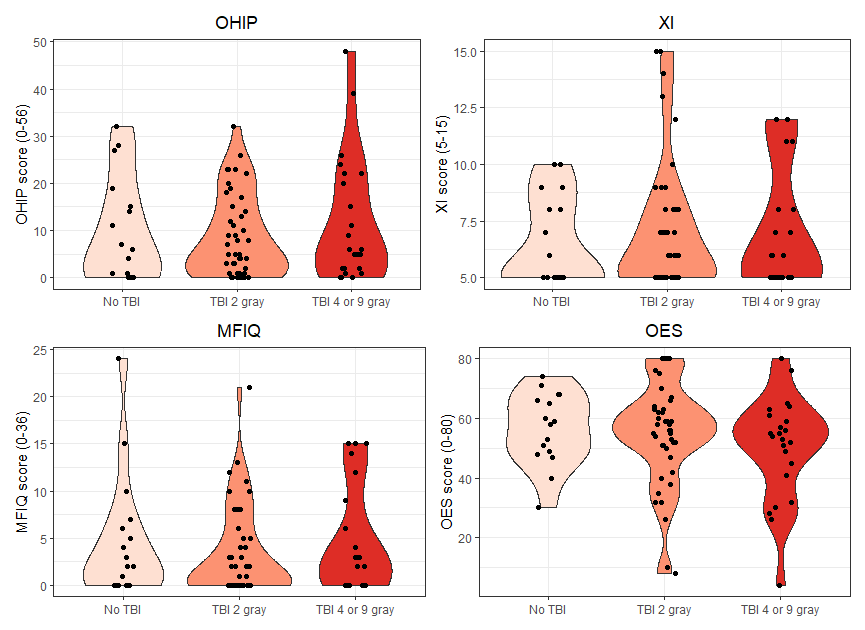
**

**Figure S7.** Violin plots with the sum scores of the OHIP-14, S-XI, MFIQ and OES questionnaires within the allogeneic HCT recipients who received to TBI, 2 Gray TBI or 4-9 Gray TBI during the conditioning therapy.


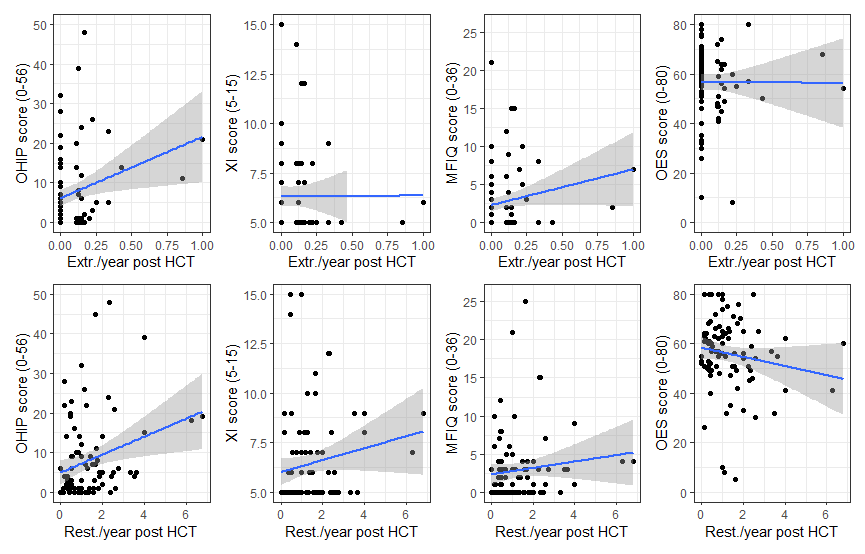


**Figure S8.** Scatterplots between the sum scores of the OHIP, XI, MFIQ and OES questionnaires and the number of extractions per year post-HCT (top row) and the number of restorations per year post-HCT (bottom row). In the top row, the patient with full mouth extractions is excluded.
